# How to simulate dissociative chemisorption of methane on metal surfaces

**DOI:** 10.3389/fchem.2024.1481235

**Published:** 2024-10-09

**Authors:** Nick Gerrits

**Affiliations:** Gorlaeus Laboratories, Leiden Institute of Chemistry, Leiden University, Leiden, Netherlands

**Keywords:** heterogeneous catalysis, density functional theory, surface science, dissociative chemisorption, methane, metal surfaces, theoretical chemistry, chemical reactivity

## Abstract

The dissociation of methane is not only an important reaction step in catalytic processes, but also of fundamental interest. Dynamical effects during the dissociative chemisorption of methane on metal surfaces cause significant differences in computed reaction rates, compared to what is predicted by typical transition state theory (TST) models. It is clear that for a good understanding of the catalytic activation of methane dynamical simulations are required. In this paper, a general blueprint is provided for performing dynamical simulations of the dissociative chemisorption of methane on metal surfaces, by employing either the quasi-classical trajectory or ring polymer molecular dynamics approach. If the computational setup is constructed with great care–since results can be affected considerably by the setup – chemically accurate predictions are achievable. Although this paper concerns methane dissociation, the provided blueprint is, so far, applicable to the dissociative chemisorption of most molecules.

## 1 Introduction

Methane steam reforming is an important industrial process to produce syngas, where the dissociative chemisorption (DC) of methane (i.e., breaking the first CH bond) is typically the rate controlling step (Wei and Iglesia, 2004; Zhang et al., 2021). Unfortunately, methane dissociation is a highly activated catalytic reaction, requiring a large amount of energy in the form of high temperature and pressure. In order to meet future sustainability goals, the energy consumption of methane activation needs to be reduced. Therefore, simulations of the DC of the methane molecule on a metal surface are not just of fundamental interest, but also practical.

In surface science, single crystal surface facets are investigated instead of real catalytic surfaces that exhibit many different facets. The reduced complexity helps gaining a clear understanding of the surface properties and how they affect molecule-surface interactions, while still being valuable for understanding heterogeneous catalysis (Ertl, 1983; Ertl, 1990). Such investigations are performed experimentally using, e.g., supersonic molecular beams, with very accurate Miller indexed cuts through the metal that ensure a defect rate lower than 0.1% (Kroes, 2021). For molecular beam studies of DC, defects that are extremely more reactive or are highly accessible through mobile trapping of the molecule rarely affect the results significantly. Additionally, so-called stepped instead of flat single crystal surfaces can often yield good understanding of defects. Although, sometimes it is necessary to simulate considerably large unit cell sizes to accurately represent catalytic materials (Imbihl et al., 2007; Gerrits, 2021a).

Unfortunately, the success of atomistic theoretical simulations hinges on many factors, e.g., the electronic structure theory, the (dynamical) model, and the tractability. For the electronic structure, density functional theory (DFT) is the workhorse method of choice, but which density functional (DF) to employ is not straightforward (Kroes, 2021; Díaz et al., 2009; Nattino et al., 2016a; Nattino et al., 2016b; Migliorini et al., 2017; Gerrits et al., 2020a). For example, if the difference between the surface’ work function and molecule’s electron affinity is smaller than 7 eV, all generalized gradient approximation (GGA) DFs are expected to underestimate the barrier height (Gerrits et al., 2020a). Fortunately, for methane, this difference is typically much larger than the threshold of 7 eV, generally allowing the use of affordable GGA DFs. But even then, not any GGA DF can be employed (Nattino et al., 2016a; Nattino et al., 2016b; Migliorini et al., 2017; Chadwick et al., 2018a; Tchakoua et al., 2023).

The employed (dynamical) model is also very important as it can have large consequences for the determination of reaction rates. For example, molecular dynamics (MD) simulations of the DC of ${\text{CHD}}_{3}$ on Pt(111) using two potential energy surfaces (PESs) obtained with different DFs (PBE (Perdew et al., 1996) and SRP32-vdW-DF1 (Nattino et al., 2016b)) yield a difference of 13 kJ/mol in the sticking probability $S_{0}$ (Chadwick et al., 2018a), which is an indirect measure of the barrier heights being crossed. However, the minimum barrier height and geometry yielded by the two PESs are nearly identical, suggesting that TST models, which often rely on a single barrier and the shape of the PES surrounding it, are inadequate for the prediction of reaction rates, at least for the DC of methane. Indeed, dynamical effects arising from traversing the PES prior to reaching the transition state (TS), where it is not even certain that the molecule manages to get close to the minimum TS, have been shown to greatly influence the reactivity of methane on metal surfaces (Gerrits et al., 2019a). Generally, if the barrier height is large and the system can be classified as a late barrier system (i.e., the dissociating bond is extended considerably at the TS), the bobsled effect (Marcus, 1966; McCullough and Wyatt, 1969) causes the molecule to slide off the minimum energy path (MEP) when it is trying to “round the corner” on the PES (Polanyi, 1972). Subsequently, the molecule needs to overcome a larger barrier height, effectively lowering the reactivity, which is typically observed for methane (Gerrits et al., 2019a; Gerrits et al., 2018; Gerrits et al., 2019b). For this reason, vibrational excitation of methane has often been observed to be relatively more effective at promoting reactivity than increasing the translational energy, if the excited vibrational mode (partially) aligns with the reaction coordinate at the TS (Nattino et al., 2016b; Migliorini et al., 2017; Gerrits et al., 2019a; Gerrits et al., 2019b; Verhoef et al., 1993; Higgins et al., 2001; Beck et al., 2003; Juurlink et al., 2009; Bisson et al., 2010; Jackson et al., 2014; Hundt et al., 2015). Furthermore, the minimum barrier height of methane on Cu(211) (i.e., a stepped surface which serves as a model for catalyst defects) is ∼ 30 kJ/mol lower than on the flat Cu(111) surface, even though *ab initio* molecular dynamics (AIMD) simulations yield similar sticking probabilities for the two surface facets (Gerrits et al., 2018). Analysis of the dynamics and the PES suggests that although the minimum barrier height is much lower and locally increases the reactivity, other parts of the surface become less reactive, overall leading to a similar reactivity when comparing the two surface facets. This again illustrates the importance of dynamical effects in determining reaction rates.

Finally, AIMD simulations are expensive, because they often require 500–2000 on-the-fly DFT calculations per trajectory (one DFT calculation per time step) under the conditions typically simulated. Moreover, if one compares to $S_{0}\in \left[0.01\ldots 0.99\right]$ measured with the King and Wells approach (King and Wells, 1972) using supersonic molecular beams (i.e., the experimental gold standard), 500–2000 MD trajectories are required to obtain errorbars of similar size (Kroes, 2021; Migliorini et al., 2017). For each order of magnitude reduction in $S_{0}$ , about 10−100× more trajectories are required to obtain relative error margins similar to the experiments. Clearly, the tractability limits AIMD studies in the amount of simulated initial conditions, and requires access to large-scale high-performance computing infrastructure that is not widely available to computational chemists. Precomputing the PES is a convenient way of saving computational costs, since the majority of all trajectories samples roughly the same parts of the PES. Many approaches to fitting or interpolating PESs exist, for which I refer the reader to other literature. One notable approach is the high-dimensional neural network potential approach by Behler and Parrinello (Behler and Parrinello, 2007). To the best of my knowledge, this is the only chemically accurate fitting approach (i.e., the mean fitting error of the entire system is lower than 4.2 kJ/mol) so far applied to the DC of methane, that also explicitly includes surface atom motion (Gerrits et al., 2019a; Gerrits et al., 2024). The latter point is critical for simulations of methane, because surface atom motion has a large effect on the reactivity, especially under catalytic conditions (Gerrits et al., 2019a; Gerrits et al., 2019b; Gerrits et al., 2024; Jackson and Nave, 2013; Campbell et al., 2015; Guo et al., 2016; Zhou and Jiang, 2019; Moiraghi et al., 2020).

**FIGURE 1:**
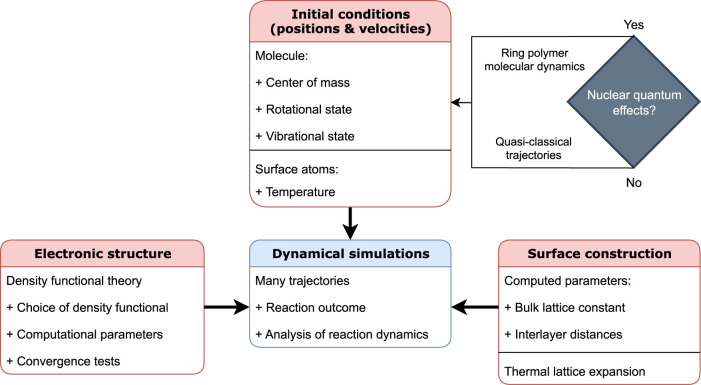
Key aspects of setting up and performing dynamical simulations of DC of molecules on surfaces. The three main categories that are needed as “inputs” are the electronic structure, construction of the surface, and initial conditions (i.e., atomic positions and velocities) of both the molecule and the metal surface.

From above, it is clear that, at present, some form of MD simulations is required to accurately compute reaction rates for the DC of methane and to analyse the reaction mechanism. Unfortunately, many non-trivial considerations go into setting up such calculations, which can influence the computed reactivity considerably. Therefore, in this paper, I will discuss what choices need to be made and why (with a focus on the quasi-classical trajectory (QCT) approach), as well as provide a blueprint for future dynamical simulations of methane. The key aspects of setting up these dynamical calculations are shown in Figure 1. This blueprint is, so far, largely applicable to the activated DC of any molecule.

## 2 Choice of density functional

The choice of the DF is important, because it underpins the entire simulation and conclusions drawn from it. As mentioned above, GGA DFs should be suitable for the DC of methane. So far, the semi-empirical SRP32-vdW-DF1 DF is the only chemically accurate DF for ${\text{CHD}}_{3}$ + Ni(111) (Nattino et al., 2016b), Pt(111) (Migliorini et al., 2017), and Pt(211) (Migliorini et al., 2017), and is likely accurate for Pd(111) as well (Gerrits et al., 2019b). Similarly, the SBH17 database ranks this DF as the best for the tested methane reactions (Tchakoua et al., 2023). In this DF, a linear combination of 32% RPBE (Hammer et al., 1999) and 68% PBE (Perdew et al., 1996) exchange is used, combined with vdW-DF1 correlation (Dion et al., 2004).

Interestingly, PBE (Perdew et al., 1996) yields similar errors across the methane subset of SBH17 as SRP32-vdW-DF1,^14^ but it is likely that the PESs yielded by PBE are too reactive for methane (Chadwick et al., 2018a). The disagreement in $S_{0}$ yielded by the two DFs has been attributed to differences in the (non-) local correlation DF (Chadwick et al., 2018a), suggesting that the use of a non-local correlation DF is a necessity.

It should also be noted that SRP32-vdW-DF1 performed poorly for the DC of methane on Pt(210) and reconstructed Pt(110)- (2×1) (Chadwick et al., 2019a; Chadwick et al., 2019b), suggesting that GGA DFs perform poorly when the reactive metal atoms are considerably undercoordinated. This carries severe consequences for simulations of single-atom alloys, if the single atom is adsorbed on top of the surface, since the atom is highly undercoordinated. It is possible that due to the large undercoordination, the electron transfer and concomitant self-interaction error (SIE) are increased. Screened hybrid DFs that employ exact exchange could reduce the SIE, but are also prohibitively expensive (Gerrits et al., 2020a). Another possibility is that the undercoordinated metal atom locally gains a more molecular character, again affecting electron densities and barrier heights. A GGA DF cannot adequately distinguish such different electronic density regimes, requiring a meta-generalized gradient approximation (mGGA) DF instead (Peverati and Truhlar, 2014). Compared to SRP32-vdW-DF1, AIMD simulations for Pt(110)- (2×1) with an mGGA DF [i.e., MS-PBEl-rVV10 (Smeets et al., 2019; Smeets and Kroes, 2021)] yielded improved agreement with experiment (Wei et al., 2021). The MS-PBEl-rVV10 DF is not only an mGGA, but also contains an approximate correction to the SIE by reproducing the exact energy of a free hydrogen atom. Therefore, it is not clear where the error yielded by GGA DFs originates. A study into reaction networks for ${\text{CO}}_{2}$ hydrogenation towards methanol on Cu surfaces employing the rMS-RPBEl-rVV10 DF also concluded that such an mGGA DF yields more robust predictions for a wide variety of molecule-metal surface reactions (Cai et al., 2024).

In short, when simulating the DC of methane on close-packed surfaces, where the reactive site is reasonably coordinated, I advice to use SRP32-vdW-DF1, because it will usually yield chemically accurate results. When the reactive site involves a metal atom that is considerably undercoordinated, a more advanced DF is required. Although more expensive than GGA (roughly a factor 3 Mejia-Rodriguez and Trickey, 2008), mGGA DFs of the MS-PBEl family seem to offer a good balance between performance and computational cost (Gerrits et al., 2020a; Tchakoua et al., 2023; Smeets et al., 2019; Smeets and Kroes, 2021; Wei et al., 2021; Cai et al., 2024; Gerrits et al., 2020b; Gerrits et al., 2021). Future efforts should establish which specific mGGA DF is a more general-purpose DF for the DC of methane. Moreover, technical advancements can bring the computational cost down, e.g., regularization of the iso-orbital indicator (Cai et al., 2024; Furness and Sun, 2019; Furness et al., 2020) and de-orbitalization to remove the expensive dependence on the kinetic energy density (Mejia-Rodriguez and Trickey, 2018; Mejia-Rodriguez and Trickey, 2017; Tran et al., 2018; Mejía-Rodríguez and Trickey, 2020). Hopefully, developments will also make the use of exact exchange tractable.

## 3 Geometries

Although dynamical simulations are a necessity in order to compute (chemically) accurate reaction probabilities, properties extracted from the PES with static calculations can still provide valuable insights. When computing static PES properties, one needs to be aware of the precise reaction mechanism that is at play. Since the DC of methane is a highly activated process, the reaction proceeds typically directly from the gas phase towards the TS at the surface, without prior physisorption or (thermal) equilibration. Although it should be noted that precursor mediated reaction of methane has also been observed (Moiraghi et al., 2020; Seets et al., 1997a; Seets et al., 1997b). This means that the asymptotic value used to compute the barrier height (since the barrier height is a relative energy) corresponds to gaseous ${\text{CH}}_{4}$ (g), and not physisorbed ${\text{CH}}_{4}$ *. Erroneously, the latter is often used, instead of the former. Unfortunately, this might lead to considerable errors in computed reaction rates employed in, e.g., microkinetic models. Furthermore, since the reaction is typically direct and rapid, surface atoms do not have the time to relax towards the DC TS (Gerrits et al., 2019a). Thus, surface atoms should be kept fixed at their ideal positions in TS search calculations, i.e., only the molecular degrees of freedom (DOFs) should be optimized. To investigate the effect of surface atom motion on the TS, the nearest surface atom can be slightly displaced along the surface normal. Subsequently, the surface DOFs are kept frozen and the TS is re-optimized. From the barrier height and geometry, the electronic and mechanical coupling can be obtained, which are indicative of barrier height and geometry changes associated with surface atom motion (Nave et al., 2014).

Moreover, the surface needs to be treated with care, as it affects results considerably (Tchakoua et al., 2023; Mondal et al., 2013). The bulk lattice constant should be obtained with a similar computational setup to the rest of the calculations. With the computed bulk lattice constant, a slab with specific Miller indices can be constructed. Subsequently, the interlayer distances are optimized, where the bottom interlayer distances are often fixed to their bulk values, in order to retain a bulk-like behaviour, even if the slab is rather thin. The rule of thumb is to leave at least the top three layers mobile. If the simulated surface temperature is non-zero, as it should be in dynamical simulations of methane, the lattice is expanded in all directions with the experimental thermal expansion coefficient (Mondal et al., 2013).

Obviously, the convergence of, e.g., the number of layers, supercell size, and vacuum distance needs to be validated. A typical benchmark is to compute the TS geometry with a reasonable computational setup and the dimer method (Henkelman and Jónsson, 1999). The resulting geometry is then used in single point calculations using different computational setups to gauge the convergence. Of course, other parameters than the aforementioned ones can be checked this way as well. From personal experience, the following parameters are generally the bare minimum if chemical accuracy (i.e., an error lower than 4.2 kJ/mol) is desired: 4 surface layers, supercell size in the X and Y direction of at least 7Å, 13Å vacuum distance between the slabs, a Γ -centered 6×6×1k -point grid, and a kinetic energy cut-off of 400 eV (assuming projector augmented wave pseudopotentials of a similar accuracy as those provided with VASP (Kresse and Joubert, 1999); also note that forces need a slightly higher cut-off than energies for convergence, even when employing support grids for more accurate forces).

It should be emphasized that a vacuum distance of 13Å is not converged for methane when employing a non-local correlation DF (as one should, *vide supra*). Considerably larger vacuum gaps are required, but are computationally more expensive: Even though the vacuum is empty, a larger distance between the slabs still yields a larger computational cost, because it scales with the real-space system size. Typically, the error is about 2–5 kJ/mol for a vacuum distance of 13Å and only dependents on the maximum distance between the periodic slabs and molecule. Thus, a common trick in MD simulations of reactive scattering is to compute the error in the minimum barrier height due to the vacuum distance not being converged. Then, to compensate, the error in the barrier height is added to the initial incidence energy of the molecule (Nattino et al., 2016b).

## 4 Dynamical simulations

The dynamical simulations can be performed with the QCT approach. In this approach, microcanonical ( NVE ) calculations are performed by propagating Newton’s equations of motion, using the atomic forces of the system. For each QCT, the reactive scattering of a single molecule from a well-defined surface is simulated. For the initial conditions of the dynamical simulations, 4 main DOFs can be distinguished: Center of mass, rotational state, vibrational state, and surface atom motion. Here, I will discuss how one might obtain these initial conditions, and how to define the reaction outcome.

### 4.1 Center of mass

For the center of mass, the most straightforward approach is to simulate only a single incidence energy $E_{\text{i}}$ for each data point, i.e., a so-called monochromatic molecular beam. However, in experiments, the incidence energy is a distribution, which can affect the sticking probability considerably (Kroes, 2021). This distribution can be measured with time-of-flight techniques and is typically fitted with the following flux-weighted velocity distribution (in the case of a supersonic molecular beam) (Michelsen and Auerbach, 1991):

$$f_{\text{i}}\left(v_{\text{i}}\right)=Av_{\text{i}}^{3}\exp\left[\frac{-{\left(v_{\text{i}}-v_{0}\right)}^{2}}{\alpha^{2}}\right],$$ (1)

where $v_{0}$ and α are the fitted stream velocity and velocity width parameters, respectively, and A is the normalization constant. Alternatively, one can use the expression for the energy distribution. It should be emphasized that $\frac{1}{2}m\alpha \neq \Delta E_{0}$ and $\frac{1}{2}m{\left(v_{\text{i}}-v_{0}\right)}^{2}\neq \left(E_{\text{i}}-E_{0}\right)$ , because the velocities are relative instead of absolute. Unfortunately, this incorrect assumption has occasionally been employed and has led to incorrect equations in literature (Holmblad et al., 1995; Larsen et al., 1999; Bukoski et al., 2003; Bernard and Harrison, 2024). Instead, the following equation is the correct flux-weighted energy distribution, analogous to Equation 1:

$$f_{\text{i}}\left(E_{\text{i}}\right)=NE_{\text{i}}\exp\left[-4E_{0}\frac{{\left(\sqrt{E_{\text{i}}}-\sqrt{E_{0}}\right)}^{2}}{{\left(\Delta E_{0}\right)}^{2}}\right],$$ (2)

$$N={\left(\frac{\Delta E_{0}}{2S}\right)}^{2}\left\{\left(1+S^{2}\right)\exp\left(-S^{2}\right)+\sqrt{\pi }\left(3/2+S^{2}\right)\left[1+\text{erf}\left(S\right)\right]\right\}$$ (3)

where $S=v_{0}/\alpha$ and $\Delta E_{0}/E_{0}\equiv 2\alpha /v_{0}$ . (Michelsen and Auerbach, 1991) Although the velocity distribution can be accurately measured and described, the experimental data is often missing in literature. Thus, molecular beam parameters are often only available through private communication or are guessed. Hopefully, in the future, it will become the norm for molecular beam experiments to include the time-of-flight spectra and data in publications.

Many experiments assume normal energy scaling (i.e., $E_{\text{n}}=\cos^{2}\left(\theta_{\text{i}}\right)E_{\text{i}}$ ), where only translational energy perpendicular to the surface promotes reactivity, and not energy parallel to the surface (Kroes, 2021). This allows the use of vibrationally hot molecular beams combined with low normal incidence energies, by varying the incidence angle instead of the temperature, seeding gas or ratio. However, the assumption of normal energy scaling should always be tested, especially if large incidence angles $\left(\theta_{\text{i}}\right)$ are employed, and generally only holds for flat surfaces and direct DC (i.e., not precursor mediated) (Gee et al., 2003; Chadwick et al., 2018b). Moreover, the choice of the azimuthal angle ϕ for off-normal incident beams is dependent on the experiment. For example, for a flat surface a uniform distribution is probably sufficient [unless finer details like diffraction are investigated (Zugarramurdi and Borisov, 2013)], but stepped surfaces often show a larger dependence on ϕ (Chadwick et al., 2018b; Salmeron et al., 1977; McMaster and Madix, 1993; Bisson et al., 2007). Finally, the position samples uniformly the supercell (XY) and is placed halfway between the two periodic slabs (Z) .

### 4.2 Rotational state

So far, rotational excitation of methane is observed to have a very limited effect on the rotational state-specific $S_{0}$ (Juurlink et al., 2000). Nevertheless, the orientation still plays an important role in the reaction dynamics, and, therefore, it is important to be able to correctly describe the initial rotational state (Gerrits et al., 2019b; Yoder et al., 2010; Nattino et al., 2014; Jackson, 2022). The orientation and momentum of the rotational state is dependent on the isotope: ${\text{CH}}_{4}$ and ${\text{CD}}_{4}$ are a spherical top rotor, ${\text{CH}}_{3}\text{D}$ and ${\text{CHD}}_{3}$ are a symmetric top rotor, and ${\text{CH}}_{2}{\text{D}}_{2}$ is an asymmetric top rotor. The state is defined by the J and M quantum numbers (Brink and Satchler, 1968). Due to the change in symmetry of the principle moments of inertia, for the symmetric top, the additional quantum number K is required, and for the asymmetric top K is split further into two different numbers.

Here, I discuss how to set up the rotational state of the symmetric top rotor ${\text{CHD}}_{3}$ (Gerrits, 2021b). For the spherical and asymmetrical top rotors, the approach is similar and can be found in literature as well (Brink and Satchler, 1968). A space fixed reference frame is employed, where the Z axis (i.e., the vector normal to the surface plane) is fixed in space. The two quantum numbers J and M define the orientation of the angular momentum vector, where J corresponds to the total rotational angular momentum L and M to its projection on the Z axis:

$$|L|=\hbar \sqrt{J\left(J+1\right)},$$ (4)

$$L_{Z}=\hbar M.$$ (5)

**FIGURE 2:**
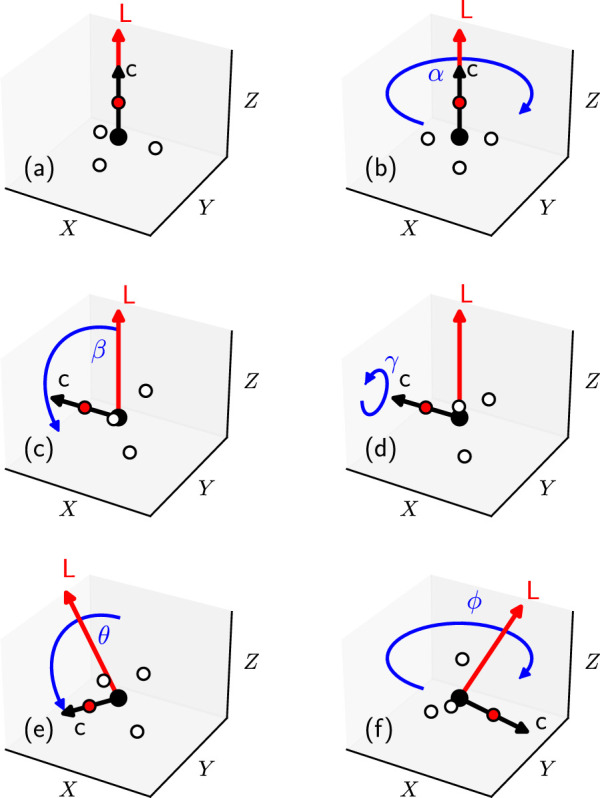
**(A)** The initial orientation of a symmetric top molecule (black arrow), here ${\text{CHD}}_{3}$ (C in black, H in red, D in white), and its angular momentum vector (red arrow) are fixed with respect to the space fixed reference frame (XYZ) . **(B–F)** Same as panel a, but indicating the rotations (blue arrows) of the molecular orientation and the angular momentum vectors required according to the quantum numbers J , M , and K . See the text for the meaning of the rotations.

Additionally, the quantum number K fixes the orientation of the figure axis (the principle axis C) with respect to the angular momentum vector:

$$L_{\text{figure}}=\hbar K,$$ (6)

and K,M∈[−J,−J+1,…,J−1,J] . The orientation of the molecule and the angular momentum vector can then be obtained as follows. First, both the figure axis C of the molecule and the angular momentum vector L are oriented parallel to the surface normal Z (Figure 2A). Then, the figure axis is rotated by the α , β , and γ Euler angles using the ZYZ convention (Figures 2B–D, respectively). The rotations by the α and γ angles are both sampled uniformly in the interval [0,2π) , whereas the angle β is computed from J and K :

$$\cos\left(\beta \right)=\frac{K}{\sqrt{J\left(J+1\right)}}.$$ (7)

Finally, both the figure axis and the angular momentum vector are rotated by the spherical θ and ϕ angles (Figures 2E, F, respectively) about the Z axis. The polar angle θ is computed from J and M :

$$\cos\left(\theta \right)=\frac{M}{\sqrt{J\left(J+1\right)}}.$$ (8)

The azimuthal angle ϕ is sampled uniformly in the interval [0,2π) . If J=0 , one can simply obtain the molecular orientation by uniformly sampling β from a $\sin\left(\beta \right)$ distribution, and α and γ from the [0,2π) interval, where the angular momentum is zero (i.e., |L|=0 ). In molecular beam simulations of methane, often only the rotational ground state is simulated, because rotational excitation does not affect the reactivity considerably, especially with the typically low rotational temperatures employed (Nattino et al., 2016b; Migliorini et al., 2017; Juurlink et al., 2000). However, this approximation might not hold if the surface corrugation and anisotropy is considerably increased, due to, e.g., surface defects.

### 4.3 Vibrational state

In QCT, the vibrational initial conditions of a molecule are obtained by micro-canonical sampling of each of its vibrational modes (Karplus et al., 1965). A 1D MD simulation is performed along each mode (i.e., the vibrational modes are not coupled), from which the initial displacement and concomitant velocity is selected by randomly sampling the phase of the vibration. Subsequently, the sum of the mode-specific displacements and velocities are added to the atomic positions and velocities, while also taking into account the orientation of the molecule given by its rotational state. The 1D potentials are computed along the normal mode Cartesian vectors extracted from the Hessian, which is obtained through finite differences. The vibrational quantum mechanical energies are determined through 1D quantum dynamics (QD) calculations on the same potential [see, e.g., Ref (Colbert and Miller, 1992)].

In principle, more accurate (semi-)classical methods can be employed to obtain the vibrational distributions, as long as it is done on the same PES as the rest of the calculations (Nguyen and Barker, 2010). But it is likely that the accuracy would mainly increase when multiple modes are simultaneously excited. Unfortunately, in this case, the QCT approach is considerably less accurate due to artificial intramolecular vibrational energy redistribution (IVR), causing overestimation of the reactivity (Gerrits et al., 2024). Multi-mode excitation of a molecule is also difficult to model, due to the mixing of modes (Hundt et al., 2017). Ring polymer molecular dynamics (RPMD) has been found to be a suitable alternative to QCT for the DC of methane on Pt(111), especially if the translational energy is low or the vibrational energy is high (Gerrits et al., 2024). Due to the approximate inclusion of nuclear quantum effects in RPMD, artificial IVR is reduced, zero-point energy (ZPE) is conserved, and tunneling effects are included. For RPMD, a large portion of the initial conditions are obtained in the same manner as for QCT, because the translational and rotational motion are only applied to the “classical” centroid, ignoring related quantum effects in the ring polymer normal modes. For the vibrational initial conditions, a different approach is required (Gerrits et al., 2024). At present, it is only possible to simulate a thermal Boltzmann distribution of the vibrational modes, instead of state-specific initial conditions. Specifically, a canonical ( NVT ) simulation of the molecule is performed, from which snapshots are taken. If a full-dimensional gas phase calculation is employed to obtain the snapshots, the translational and rotational motion are removed afterwards to retain solely the vibrational motion. If a low temperature is employed, the distribution corresponds roughly to the vibrational ground state.

So far, I have only discussed how to obtain a rovibrational state, but not its thermostatistical weight. The rovibrational state population $F_{\nu_{i},J}$ of a molecule in the molecular beam is typically given by

$$F_{\nu_{i},J}\left(T_{\text{vib}},T_{\text{rot}}\right)=\frac{2J+1}{Z\left(T_{\text{vib}},T_{\text{rot}}\right)}\exp\left(-\frac{\left(E_{\nu_{i},0}-E_{0,0}\right)}{k_{\text{B}}T_{\text{vib}}}\right)\exp\left(-\frac{\left(E_{\nu_{i},J}-E_{\nu_{i},0}\right)}{k_{\text{B}}T_{\text{rot}}}\right),$$ (9)

where $Z\left(T_{\text{vib}},T_{\text{rot}}\right)$ is the partition function, and $T_{\text{vib}}$ and $T_{\text{rot}}$ are the vibrational and rotational temperatures, respectively. It should be noted that intrapolyad cooling can cause a slight deviation of the experimental vibrational distribution from the Boltzmann distribution, but again the effect is limited and hardly affects $S_{0}$ (Nattino et al., 2016b)

### 4.4 Surface atom motion

To simulate the effect of surface temperature $\left(T_{\text{s}}\right)$ , a procedure can be employed as described in Refs Nattino et al. (2016b) and Nattino et al. (2012), which largely avoids a possibly lengthy equilibration time often associated with the usually employed NVT approach for surface atom motion. An independent 1D harmonic oscillator model is used to mimic the thermal motion of the surface atoms, by assigning initial displacements and velocities to the atoms of the mobile layers. Using $K=1/2mv^{2}$ and $U=1/2kx^{2}$ (kinetic and potential energy, respectively), the following Boltzmann distributions for the velocities and positions are sampled:

$$f\left(v\right)={\left(\frac{m}{2\pi k_{\text{B}}T}\right)}^{\frac{1}{2}}e^{\frac{-mv^{2}}{2k_{\text{B}}T}}$$ (10)

$$f\left(q\right)={\left(\frac{m\omega^{2}}{2\pi k_{\text{B}}T}\right)}^{\frac{1}{2}}e^{\frac{-m\omega^{2}q^{2}}{2k_{\text{B}}T}}$$ (11)

The frequency ω is obtained by performing normal mode calculations for each single atom (that is not symmetrically identical) in an ideal metal slab. This yields the frequencies that are employed in the aforementioned Boltzmann distribution f(q) . Furthermore, the theoretically computed lattice constant (*vide supra*) is expanded by an experimentally obtained lattice expansion coefficient, in order to account for the thermal expansion from T_s_ = 0 K to the simulated surface temperature (Mondal et al., 2013). This is achieved, effectively, by multiplying all three lattice vectors with the expansion coefficient, to also include the aforementioned relaxed interlayer distances. Several differently-initialized slabs are generated using this procedure, which are equilibrated by performing NVE calculations and allowing the atoms in the mobile layers to move in all directions. Once the surface is equilibrated, the configurations (positions and velocities) of these NVE simulations are gathered to form a pool of initial conditions. The atoms in the bottom layer(s) of the metal slab are kept fixed in their ideal positions during the calculations.

The simulated surface temperature should be above the Debye temperature, which also reduces the issue of trapped trajectories (Nattino et al., 2016b; Migliorini et al., 2017; Manson, 1991; Manson, 1994). Otherwise, classical MD yields incorrect phonon distributions. At present, it is unclear whether RPMD would correctly describe the phonon distribution at low surface temperatures. Regardless, if you use RPMD, it is advised to use NVT calculations instead, because the ring polymer normal modes are more easily converged that way, especially with specialized quantum thermostats (Ceriotti et al., 2009; Ceriotti et al., 2010; Ceriotti and Manolopoulos, 2012).

### 4.5 Reaction outcome

For dynamical simulations of the DC of methane, typically three different reaction outcomes are defined: scattering, dissociation, and trapping. Methane is often considered to be scattered when the distance between the surface macroscopic plane and methane’s center of mass is larger than half of the vacuum distance (i.e., larger than the initial condition) and its momentum is pointing away from the surface. It is possible to reduce the distance criterium to save computational cost, but it should be checked whether this affects results (e.g., how bouncing trajectories are counted). Furthermore, methane is considered to be reacted if one of the intramolecular bonds is considerably extended beyond the TS value, or a smaller length for a certain amount of time. Typically safe parameters are $r_{\text{diss}}>3\overset{{}^{\circ}}{A}$ , or $r_{\text{diss}}>2\overset{{}^{\circ}}{A}$ for 100 fs. Finally, if none of the aforementioned results are obtained within the simulation time, the molecule is considered to be trapped. It should be noted that computed trapping probabilities are always an upper limit of the experiment. Trapped trajectories might still desorb or react, but the timescale involved is considerably longer than what is tractable for theory. However, experimentally such events are often measured as scattered, thus, lowering the trapping probability compared to theory.

The reaction probability R is defined as $R=N_{\text{r}}/N_{\text{i}}$ , where $N_{\text{r}}$ and $N_{\text{i}}$ are the amount of reacted and initial trajectories, respectively. Similarly, the sticking probability $S_{0}$ , which includes contributions of both reacted and trapped trajectories, is defined as $S_{0}=\left(N_{\text{r}}+N_{\text{t}}\right)/N_{\text{i}}$ , where $N_{\text{t}}$ is the amount of trapped trajectories.

The energy transfer $E_{\text{T}}$ from methane to the metal surface can be defined as

$$E_{\text{T}}=\left(V_{\text{i}}+K_{\text{i}}\right)-\left(V_{\text{f}}+K_{\text{f}}\right),$$ (12)

where V and K are the potential electronic and kinetic energy of methane, respectively, at the initial (i) and final (f) time steps of the scattered trajectories.

Finally, if RPMD is employed, observables are generally computed in the same fashion as with QCT, by simply using the centroid.

## 5 Conclusion

The DC of methane on metal surfaces is an important reaction step in catalytic processes. Dynamical effects cause significant deviation in reaction rates and mechanisms, compared to what is predicted by TST models. Therefore, for an accurate description and understanding of the DC of methane, dynamical simulations are required. Performing such calculations is not trivial and many choices have to be made. In this paper, I have described how an accurate dynamical simulation might be set up within the QCT approach, or alternatively using RPMD. Perhaps the most important points are the choice of DF, the way the surface geometry is obtained, the dynamical model, and the construction of the initial conditions. If the dynamical calculations are carefully constructed, chemically accurate predictions are possible. Moreover, most of the choices made here are the same or similar for simulations of the DC of molecules other than methane. Therefore, this work also serves as a blueprint for simulating DC in general.

## References

[B1] Beck R. D. Maroni P. Papageorgopoulos D. C. Dang T. T. Schmid M. P. Rizzo T. R. (2003). Vibrational mode-specific reaction of methane on a nickel surface. Science 302, 98–100. 10.1126/science.1088996 14526078

[B2] Behler J. Parrinello M. (2007). Generalized neural-network representation of high-dimensional potential-energy surfaces. Phys. Rev. Lett. 98, 146401. 10.1103/physrevlett.98.146401 17501293

[B3] Bernard M. E. Harrison I. (2024). Microcanonical treatment of HCl dissociative chemisorption on Au(111): reactive dampening through inefficient translational energy coupling and an active surface. J. Chem. Phys. 160, 084702. 10.1063/5.0193675 38391017

[B4] Bisson R. Sacchi M. Beck R. D. (2010). Mode-specific reactivity of CH_4_ on Pt(110)-(1×2): the concerted role of stretch and bend excitation. Phys. Rev. B 82, 121404. 10.1103/physrevb.82.121404

[B5] Bisson R. Sacchi M. Dang T. T. Yoder B. Maroni P. Beck R. D. (2007). State-resolved reactivity of CH4(2nu_3_) on Pt(111) and Ni(111): effects of barrier height and transition state location. J. Phys. Chem. A 111, 12679–12683. 10.1021/jp076082w 17999476

[B6] Brink D. Satchler G. (1968). Angular momentum. 2nd ed. Oxford University Press.

[B7] Bukoski A. Blumling D. Harrison I. (2003). Microcanonical unimolecular rate theory at surfaces. I. Dissociative chemisorption of methane on Pt(111). J. Chem. Phys. 118, 843–871. 10.1063/1.1525803 16164362

[B8] Cai Y. Michiels R. De Luca F. Neyts E. Tu X. Bogaerts A. (2024). Improving molecule–metal surface reaction networks using the meta-generalized gradient approximation: CO_2_ hydrogenation. J. Phys. Chem. C 128, 8611–8620. 10.1021/acs.jpcc.4c01110 PMC11145648 38835935

[B9] Campbell V. L. Chen N. Guo H. Jackson B. Utz A. L. (2015). Substrate vibrations as promoters of chemical reactivity on metal surfaces. J. Phys. Chem. A 119, 12434–12441. 10.1021/acs.jpca.5b07873 26406229

[B10] Ceriotti M. Bussi G. Parrinello M. (2009). Nuclear quantum effects in solids using a colored-noise thermostat. Phys. Rev. Lett. 103, 030603. 10.1103/physrevlett.103.030603 19659261

[B11] Ceriotti M. Manolopoulos D. E. (2012). Efficient first-principles calculation of the quantum kinetic energy and momentum distribution of nuclei. Phys. Rev. Lett. 109, 100604. 10.1103/physrevlett.109.100604 23005275

[B12] Ceriotti M. Parrinello M. Markland T. E. Manolopoulos D. E. (2010). Efficient stochastic thermostatting of path integral molecular dynamics. J. Chem. Phys. 133, 124104. 10.1063/1.3489925 20886921

[B13] Chadwick H. Gutiérrez-González A. Beck R. D. Kroes G.-J. (2019a). CHD_3_ dissociation on the kinked Pt(210) surface: a comparison of experiment and theory. J. Phys. Chem. C 123, 14530–14539. 10.1021/acs.jpcc.9b03051

[B14] Chadwick H. Gutiérrez-González A. Beck R. D. Kroes G.-J. (2019b). Transferability of the SRP32-vdW specific reaction parameter functional to CHD_3_ dissociation on Pt(110)-(2x1). J. Chem. Phys. 150, 124702. 10.1063/1.5081005 30927879

[B15] Chadwick H. Gutiérrez-González A. Migliorini D. Beck R. D. Kroes G.-J. (2018b). Incident angle dependence of CHD_3_ dissociation on the stepped Pt(211) surface. J. Phys. Chem. C 122, 19652–19660. 10.1021/acs.jpcc.8b05887 PMC6120747 30197724

[B16] Chadwick H. Migliorini D. Kroes G. J. (2018a). CHD_3_ dissociation on Pt(111): a comparison of the reaction dynamics based on the PBE functional and on a specific reaction parameter functional. J. Chem. Phys. 149, 044701. 10.1063/1.5039458 30068208

[B17] Colbert D. T. Miller W. H. (1992). A novel discrete variable representation for quantum mechanical reactive scattering via the S-matrix kohn method. J. Chem. Phys. 96, 1982–1991. 10.1063/1.462100

[B18] Díaz C. Pijper E. Olsen R. A. Busnengo H. F. Auerbach D. J. Kroes G. J. (2009). Chemically accurate simulation of a prototypical surface reaction: H_2_ dissociation on Cu(111). Science 326, 832–834. 10.1126/science.1178722 19892978

[B19] Dion M. Rydberg H. Schröder E. Langreth D. C. Lundqvist B. I. (2004). Van der waals density functional for general geometries. Phys. Rev. Lett. 92, 246401. 10.1103/physrevlett.92.246401 15245113

[B20] Ertl G. (1983). Primary steps in catalytic synthesis of ammonia. J. Vac. Sci. Technol. A 1, 1247–1253. 10.1116/1.572299

[B21] Ertl G. (1990). Elementary steps in heterogeneous catalysis. Angew. Chem. Int. Ed. 29, 1219–1227. 10.1002/anie.199012191

[B22] Furness J. W. Kaplan A. D. Ning J. Perdew J. P. Sun J. (2020). Accurate and numerically efficient r2SCAN meta-generalized gradient approximation. J. Phys. Chem. Lett. 11, 8208–8215. 10.1021/acs.jpclett.0c02405 32876454

[B23] Furness J. W. Sun J. (2019). Enhancing the efficiency of density functionals with an improved iso-orbital indicator. Phys. Rev. B 99, 041119. 10.1103/physrevb.99.041119

[B24] Gee A. T. Hayden B. E. Mormiche C. Kleyn A. W. Riedmüller B. (2003). The dynamics of the dissociative adsorption of methane on Pt(533). J. Chem. Phys. 118, 3334–3341. 10.1063/1.1538184

[B25] Gerrits N. (2021a). Accurate simulations of the reaction of H_2_ on a curved Pt crystal through machine learning. J. Phys. Chem. Lett. 12, 12157–12164. 10.1021/acs.jpclett.1c03395 34918518 PMC8724818

[B26] Gerrits N. (2021b). Accurate modeling of the dynamics of dissociative chemisorption on metal surfaces. Leiden, Netherlands: Leiden University. Ph.D. thesis.

[B27] Gerrits N. Chadwick H. Kroes G.-J. (2019b). Dynamical study of the dissociative chemisorption of CHD_3_ on Pd(111). J. Phys. Chem. C 123, 24013–24023. 10.1021/acs.jpcc.9b05757 PMC6778984 31602282

[B28] Gerrits N. Geweke J. Auerbach D. J. Beck R. D. Kroes G.-J. (2021). Highly efficient activation of HCl dissociation on Au(111) via rotational preexcitation. J. Phys. Chem. Lett. 12, 7252–7260. 10.1021/acs.jpclett.1c02093 34313445 PMC8350909

[B29] Gerrits N. Geweke J. Smeets E. W. F. Voss J. Wodtke A. M. Kroes G.-J. (2020b). Closing the gap between experiment and theory: reactive scattering of HCl from Au(111). J. Phys. Chem. C 124, 15944–15960. 10.1021/acs.jpcc.0c03756

[B30] Gerrits N. Jackson B. Bogaerts A. (2024). Accurate reaction probabilities for translational energies on both sides of the barrier of dissociative chemisorption on metal surfaces. J. Phys. Chem. Lett. 15, 2566–2572. 10.1021/acs.jpclett.3c03408 38416779 PMC10926167

[B31] Gerrits N. Migliorini D. Kroes G.-J. (2018). Dissociation of CHD_3_ on Cu(111), Cu(211), and single atom alloys of Cu(111). J. Chem. Phys. 149, 224701. 10.1063/1.5053990 30553257

[B32] Gerrits N. Shakouri K. Behler J. Kroes G.-J. (2019a). Accurate probabilities for highly activated reaction of polyatomic molecules on surfaces using a high-dimensional neural network potential: CHD_3_ + Cu(111). J. Phys. Chem. Lett. 10, 1763–1768. 10.1021/acs.jpclett.9b00560 30922058 PMC6477808

[B33] Gerrits N. Smeets E. W. F. Vuckovic S. Powell A. D. Doblhoff-Dier K. Kroes G.-J. (2020a). Density functional theory for molecule–metal surface reactions: when does the generalized gradient approximation get it right, and what to do if it does not. J. Phys. Chem. Lett. 11, 10552–10560. 10.1021/acs.jpclett.0c02452 33295770 PMC7751010

[B34] Guo H. Farjamnia A. Jackson B. (2016). Effects of lattice motion on dissociative chemisorption: toward a rigorous comparison of theory with molecular beam experiments. J. Phys. Chem. Lett. 7, 4576–4584. 10.1021/acs.jpclett.6b01948 27791370

[B35] Hammer B. Hansen L. B. Nørskov J. K. (1999). Improved adsorption energetics within density-functional theory using revised perdew-burke-ernzerhof functionals. Phys. Rev. B 59, 7413–7421. 10.1103/physrevb.59.7413

[B36] Henkelman G. Jónsson H. (1999). A dimer method for finding saddle points on high dimensional potential surfaces using only first derivatives. J. Chem. Phys. 111, 7010–7022. 10.1063/1.480097

[B37] Higgins J. Conjusteau A. Scoles G. Bernasek S. L. (2001). State selective vibrational (2*v* _3_) activation of the chemisorption of methane on Pt(111). J. Chem. Phys. 114, 5277–5283. 10.1063/1.1349895

[B38] Holmblad P. M. Wambach J. Chorkendorff I. (1995). Molecular beam study of dissociative sticking of methane on Ni(100). J. Chem. Phys. 102, 8255–8263. 10.1063/1.468955

[B39] Hundt P. M. Ueta H. van Reijzen M. E. Jiang B. Guo H. Beck R. D. (2015). Bond-selective and mode-specific dissociation of CH_3_D and CH_2_D_2_ on Pt(111). J. Phys. Chem. A 119, 12442–12448. 10.1021/acs.jpca.5b07949 26414099

[B40] Hundt P. M. van Reijzen M. E. Beck R. D. Guo H. Jackson B. (2017). Quantum-state-resolved reactivity of overtone excited CH_4_ on Ni(111): comparing experiment and theory. J. Chem. Phys. 146, 054701. 10.1063/1.4975025 28178793

[B41] Imbihl R. Behm R. Schlögl R. (2007). Bridging the pressure and material gap in heterogeneous catalysis. Phys. Chem. Chem. Phys. 9, 3459. 10.1039/b706675a 17612713

[B42] Jackson B. (2022). Quantum studies of methane-metal inelastic diffraction and trapping: the variation with molecular orientation and phonon coupling. Chem. Phys. 559, 111516. 10.1016/j.chemphys.2022.111516

[B43] Jackson B. Nattino F. Kroes G.-J. (2014). Dissociative chemisorption of methane on metal surfaces: tests of dynamical assumptions using quantum models and *ab initio* molecular dynamics. J. Chem. Phys. 141, 054102. 10.1063/1.4891327 25106565

[B44] Jackson B. Nave S. (2013). The dissociative chemisorption of methane on Ni(111): the effects of molecular vibration and lattice motion. J. Chem. Phys. 138, 174705. 10.1063/1.4802008 23656150

[B45] Juurlink L. B. Smith R. R. Utz A. L. (2000). The role of rotational excitation in the activated dissociative chemisorption of vibrationally excited methane on Ni(100). Faraday Discuss. 117, 147–160. 10.1039/b003708g 11271989

[B46] Juurlink L. B. F. Killelea D. R. Utz A. L. (2009). State-resolved probes of methane dissociation dynamics. Prog. Surf. Sci. 84, 69–134. 10.1016/j.progsurf.2009.01.001

[B47] Karplus M. Porter R. N. Sharma R. D. (1965). Exchange reactions with activation energy. I. Simple barrier potential for (H, H_2_). J. Chem. Phys. 43, 3259–3287. 10.1063/1.1697301

[B48] King D. A. Wells M. G. (1972). Molecular beam investigation of adsorption kinetics on bulk metal targets: nitrogen on tungsten. Surf. Sci. 29, 454–482. 10.1016/0039-6028(72)90232-4

[B49] Kresse G. Joubert D. (1999). From ultrasoft pseudopotentials to the projector augmented-wave method. Phys. Rev. B 59, 1758–1775. 10.1103/physrevb.59.1758

[B50] Kroes G.-J. (2021). Computational approaches to dissociative chemisorption on metals: towards chemical accuracy. Phys. Chem. Chem. Phys. 23, 8962–9048. 10.1039/d1cp00044f 33885053

[B51] Larsen J. H. Holmblad P. M. Chorkendorff I. (1999). Dissociative sticking of CH_4_ on Ru(0001). J. Chem. Phys. 110, 2637–2642. 10.1063/1.477985

[B52] Manson J. R. (1991). Inelastic scattering from surfaces. Phys. Rev. B 43, 6924–6937. 10.1103/physrevb.43.6924 9998154

[B53] Manson J. R. (1994). Multiphonon atom-surface scattering. Comput. Phys. Commun. 80, 145–167. 10.1016/0010-4655(94)90101-5

[B54] Marcus R. A. (1966). On the analytical mechanics of chemical reactions. Quantum mechanics of linear collisions. J. Chem. Phys. 45, 4493–4499. 10.1063/1.1727528

[B55] McCullough E. A. Wyatt R. E. (1969). Quantum dynamics of the collinear (H, H_2_) reaction. J. Chem. Phys. 51, 1253–1254. 10.1063/1.1672133

[B56] McMaster M. C. Madix R. J. (1993). Alkane dissociation dynamics on Pt(110)–(1×2). J. Chem. Phys. 98, 9963–9976. 10.1063/1.464322

[B57] Mejia-Rodriguez D. Trickey S. B. (2017). Deorbitalization strategies for meta-generalized-gradient-approximation exchange-correlation functionals. Phys. Rev. A 96, 052512. 10.1103/physreva.96.052512

[B58] Mejia-Rodriguez D. Trickey S. B. (2018). Deorbitalized meta-GGA exchange-correlation functionals in solids. Phys. Rev. B 98, 115161. 10.1103/physrevb.98.115161

[B59] Mejía-Rodríguez D. Trickey S. B. (2020). Meta-GGA performance in solids at almost GGA cost. Phys. Rev. B 102, 121109. 10.1103/physrevb.102.121109

[B60] Michelsen H. A. Auerbach D. J. (1991). A critical examination of data on the dissociative adsorption and associative desorption of hydrogen at copper surfaces. J. Chem. Phys. 94, 7502–7520. 10.1063/1.460182

[B61] Migliorini D. Chadwick H. Nattino F. Gutiérrez-González A. Dombrowski E. High E. A. (2017). Surface reaction barriometry: methane dissociation on flat and stepped transition-metal surfaces. J. Phys. Chem. Lett. 8, 4177–4182. 10.1021/acs.jpclett.7b01905 28817773 PMC5592645

[B62] Moiraghi R. Lozano A. Peterson E. Utz A. Dong W. Busnengo H. F. (2020). Nonthermalized precursor-mediated dissociative chemisorption at high catalysis temperatures. J. Phys. Chem. Lett. 11, 2211–2218. 10.1021/acs.jpclett.0c00260 32073863

[B63] Mondal A. Wijzenbroek M. Bonfanti M. Díaz C. Kroes G.-J. (2013). Thermal lattice expansion effect on reactive scattering of H_2_ from Cu(111) at t_s_ = 925 K. J. Phys. Chem. A 117, 8770–8781. 10.1021/jp4042183 23763274

[B64] Nattino F. Díaz C. Jackson B. Kroes G.-J. (2012). Effect of surface motion on the rotational quadrupole alignment parameter of D_2_ reacting on Cu(111). Phys. Rev. Lett. 108, 236104. 10.1103/physrevlett.108.236104 23003976

[B65] Nattino F. Migliorini D. Bonfanti M. Kroes G.-J. (2016a). Methane dissociation on Pt(111): searching for a specific reaction parameter density functional. J. Chem. Phys. 144, 044702. 10.1063/1.4939520 26827223

[B66] Nattino F. Migliorini D. Kroes G.-J. Dombrowski E. High E. A. Killelea D. R. (2016b). Chemically accurate simulation of a polyatomic molecule-metal surface reaction. J. Phys. Chem. Lett. 7, 2402–2406. 10.1021/acs.jpclett.6b01022 27284787 PMC4939468

[B67] Nattino F. Ueta H. Chadwick H. van Reijzen M. E. Beck R. D. Jackson B. (2014). *Ab initio* molecular dynamics calculations versus quantum-state-resolved experiments on CHD_3_ + Pt(111): new insights into a prototypical gas–surface reaction. J. Phys. Chem. Lett. 5, 1294–1299. 10.1021/jz500233n 26269970

[B68] Nave S. Tiwari A. K. Jackson B. (2014). Dissociative chemisorption of methane on Ni and Pt surfaces: mode-specific chemistry and the effects of lattice motion. J. Phys. Chem. A 118, 9615–9631. 10.1021/jp5063644 25153478

[B69] Nguyen T. L. Barker J. R. (2010). Sums and densities of fully coupled anharmonic vibrational states: a comparison of three practical methods. J. Phys. Chem. A 114, 3718–3730. 10.1021/jp100132s 20170143

[B70] Perdew J. P. Burke K. Ernzerhof M. (1996). Generalized gradient approximation made simple. Phys. Rev. Lett. 77, 3865–3868. 10.1103/physrevlett.77.3865 10062328

[B71] Peverati R. Truhlar D. G. (2014). Quest for a universal density functional: the accuracy of density functionals across a broad spectrum of databases in chemistry and physics. Philos. Trans. R. Soc. A 372, 20120476. 10.1098/rsta.2012.0476 24516178

[B72] Polanyi J. C. (1972). Concepts in reaction dynamics. Acc. Chem. Res. 5, 161–168. 10.1021/ar50053a001

[B73] Salmeron M. Gale R. J. Somorjai G. A. (1977). Molecular beam study of the H_2_–D_2_ exchange reaction on stepped platinum crystal surfaces: dependence on reactant angle of incidence. J. Chem. Phys. 67, 5324–5334. 10.1063/1.434711

[B74] Seets D. C. Reeves C. T. Ferguson B. A. Wheeler M. C. Mullins C. B. (1997b). Dissociative chemisorption of methane on Ir(111): evidence for direct and trapping-mediated mechanisms. J. Chem. Phys. 107, 10229–10241. 10.1063/1.475306

[B75] Seets D. C. Wheeler M. C. Mullins C. B. (1997a). Trapping-mediated and direct dissociative chemisorption of methane on Ir(110): a comparison of molecular beam and bulb experiments. J. Chem. Phys. 107, 3986–3998. 10.1063/1.474754

[B76] Smeets E. W. Voss J. Kroes G.-J. (2019). Specific reaction parameter density functional based on the meta-generalized gradient approximation: application to H_2_ + Cu(111) and H_2_ + Ag(111). J. Phys. Chem. A 123, 5395–5406. 10.1021/acs.jpca.9b02914 31149824 PMC6600505

[B77] Smeets E. W. F. Kroes G.-J. (2021). Performance of made simple meta-GGA functionals with rVV10 nonlocal correlation for H_2_ + Cu(111), D_2_ + Ag(111), H_2_ + Au(111), and D_2_ + Pt(111). J. Phys. Chem. C 125, 8993–9010. 10.1021/acs.jpcc.0c11034 PMC8162760 34084265

[B78] Tchakoua T. Gerrits N. Smeets E. W. F. Kroes G.-J. (2023). SBH17: benchmark database of barrier heights for dissociative chemisorption on transition metal surfaces. J. Chem. Theory Comput. 19, 245–270. 10.1021/acs.jctc.2c00824 36529979 PMC9835835

[B79] Tran F. Kovács P. Kalantari L. Madsen G. K. H. Blaha P. (2018). Orbital-free approximations to the kinetic-energy density in exchange-correlation MGGA functionals: tests on solids. J. Chem. Phys. 149, 144105. 10.1063/1.5048907 30316291

[B80] Verhoef R. W. Kelly D. Mullins C. B. Weinberg W. H. (1993). Isotope effects for the direct dissociative chemisorption of methane and ethane on Ir(110) and vibrationally assisted chemisorption. Surf. Sci. 287–288, A370–A398. 10.1016/0167-2584(93)90398-3

[B81] Wei F. Smeets E. W. Voss J. Kroes G.-J. Lin S. Guo H. (2021). Assessing density functionals for describing methane dissociative chemisorption on Pt(110)-(2×1) surface. Chin. J. Chem. Phys. 34, 883–895. 10.1063/1674-0068/cjcp2110207

[B82] Wei J. Iglesia E. (2004). Mechanism and site requirements for activation and chemical conversion of methane on supported Pt clusters and turnover rate comparisons among noble metals. J. Phys. Chem. B 108, 4094–4103. 10.1021/jp036985z

[B83] Yoder B. L. Bisson R. Beck R. D. (2010). Steric effects in the chemisorption of vibrationally excited methane on Ni(100). Science 329, 553–556. 10.1126/science.1191751 20671185

[B84] Zhang H. Sun Z. Hu Y. H. (2021). Steam reforming of methane: current states of catalyst design and process upgrading. Renew. Sustain. Energy Rev. 149, 111330. 10.1016/j.rser.2021.111330

[B85] Zhou X. Jiang B. (2019). A modified generalized Langevin oscillator model for activated gas-surface reactions. J. Chem. Phys. 150, 024704. 10.1063/1.5078541 30646703

[B86] Zugarramurdi A. Borisov A. G. (2013). Theoretical study of the effect of beam misalignment in fast-atom diffraction at surfaces. Phys. Rev. A 87, 062902. 10.1103/physreva.87.062902

